# Consensus recommendations for the treatment and management of patients with Fabry disease on migalastat: a modified Delphi study

**DOI:** 10.3389/fmed.2023.1220637

**Published:** 2023-09-01

**Authors:** Daniel G. Bichet, Robert J. Hopkin, Patrício Aguiar, Sridhar R. Allam, Yin-Hsiu Chien, Roberto Giugliani, Staci Kallish, Sabina Kineen, Olivier Lidove, Dau-Ming Niu, Iacopo Olivotto, Juan Politei, Paul Rakoski, Roser Torra, Camilla Tøndel, Derralynn A. Hughes

**Affiliations:** ^1^Department of Medicine, Pharmacology and Physiology, Hôpital du Sacré-Coeur, University of Montréal, Montreal, QC, Canada; ^2^Department of Pediatrics, Division of Human Genetics, University of Cincinnati College of Medicine, and Cincinnati Children’s Hospital Medical Center, Cincinnati, OH, United States; ^3^Inborn Errors of Metabolism Reference Center, Centro Hospitalar Universitário Lisboa Norte, Lisbon, Portugal; ^4^Faculty of Medicine, Lisbon University, Lisbon, Portugal; ^5^Burnett School of Medicine, Texas Christian University, Fort Worth, TX, United States; ^6^Tarrant Nephrology Associates/PPG Health, Fort Worth, TX, United States; ^7^Department of Medical Genetics, National Taiwan University Hospital, Taipei, Taiwan; ^8^Department of Pediatrics, National Taiwan University College of Medicine, Taipei, Taiwan; ^9^Postgraduate Program in Genetics and Molecular Biology (PPGBM) at Federal University of Rio Grande do Sul (UFRGS), Porto Alegre, Brazil; ^10^BioDiscovery Laboratory at Hospital de Clinicas de Porto Alegre (HCPA), National Institute of Population Medical Genetics (INAGEMP), DASA, Casa dos Raros, Porto Alegre, Brazil; ^11^Division of Translational Medicine and Human Genetics, Department of Medicine, University of Pennsylvania, Philadelphia, PA, United States; ^12^Patient Advocate, United States; ^13^Department of Internal Medicine-Rheumatology, Croix Saint Simon Hospital, Paris, France; ^14^French Network of Inherited Metabolic Disorders (G2m), France; ^15^Department of Pediatrics, Taipei Veterans General Hospital, Taipei, Taiwan; ^16^Institute of Clinical Medicine, National Yang Ming Chiao Tung University, Taipei, Taiwan; ^17^Department of Experimental and Clinical Medicine, Meyer University Children’s Hospital, Florence, Italy; ^18^Department of Neurology, Fundacion Para el Estudio de Enfermedades Neurometabolicas (FESEN), Buenos Aires, Argentina; ^19^Inherited Kidney Disorders, Department of Nephrology, Fundació Puigvert, Institut d’Investigació Biomèdica Sant Pau (IIB-SANT PAU), Universitat Autònoma de Barcelona, Barcelona, Spain; ^20^Department of Clinical Science, University of Bergen, Bergen, Norway; ^21^Department of Pediatrics, Haukeland University Hospital, Bergen, Norway; ^22^Lysosomal Storage Disorders Unit, Royal Free London NHS Foundation Trust and University College London, London, United Kingdom

**Keywords:** chaperone therapy, alpha-galactosidase A, globotriaosylsphingosine, amenability, treatment decisions, patient journey

## Abstract

**Objective:**

Fabry disease is a progressive disorder caused by deficiency of the α-galactosidase A enzyme (α-Gal A), leading to multisystemic organ damage with heterogenous clinical presentation. The addition of the oral chaperone therapy migalastat to the available treatment options for Fabry disease is not yet universally reflected in all treatment guidelines. These consensus recommendations are intended to provide guidance for the treatment and monitoring of patients with Fabry disease receiving migalastat.

**Methods:**

A modified Delphi process was conducted to determine consensus on treatment decisions and monitoring of patients with Fabry disease receiving migalastat. The multidisciplinary panel comprised 14 expert physicians across nine specialties and two patients with Fabry disease. Two rounds of Delphi surveys were completed and recommendations on the use of biomarkers, multidisciplinary monitoring, and treatment decisions were generated based on statements that reached consensus.

**Results:**

The expert panel reached consensus agreement on 49 of 54 statements, including 16 that reached consensus in round 1. Statements that reached consensus agreement are summarized in recommendations for migalastat treatment and monitoring, including baseline and follow-up assessments and frequency. All patients with Fabry disease and an amenable mutation may initiate migalastat treatment if they have evidence of Fabry-related symptoms and/or organ involvement. Treatment decisions should include holistic assessment of the patient, considering clinical symptoms and organ involvement as well as patient-reported outcomes and patient preference. The reliability of α-Gal A and globotriaosylsphingosine as pharmacodynamic response biomarkers remains unclear.

**Conclusion:**

These recommendations build on previously published guidelines to highlight the importance of holistic, multidisciplinary monitoring for patients with Fabry disease receiving migalastat, in addition to shared decision-making regarding treatments and monitoring throughout the patient journey.

**GRAPHICAL ABSTRACT:**
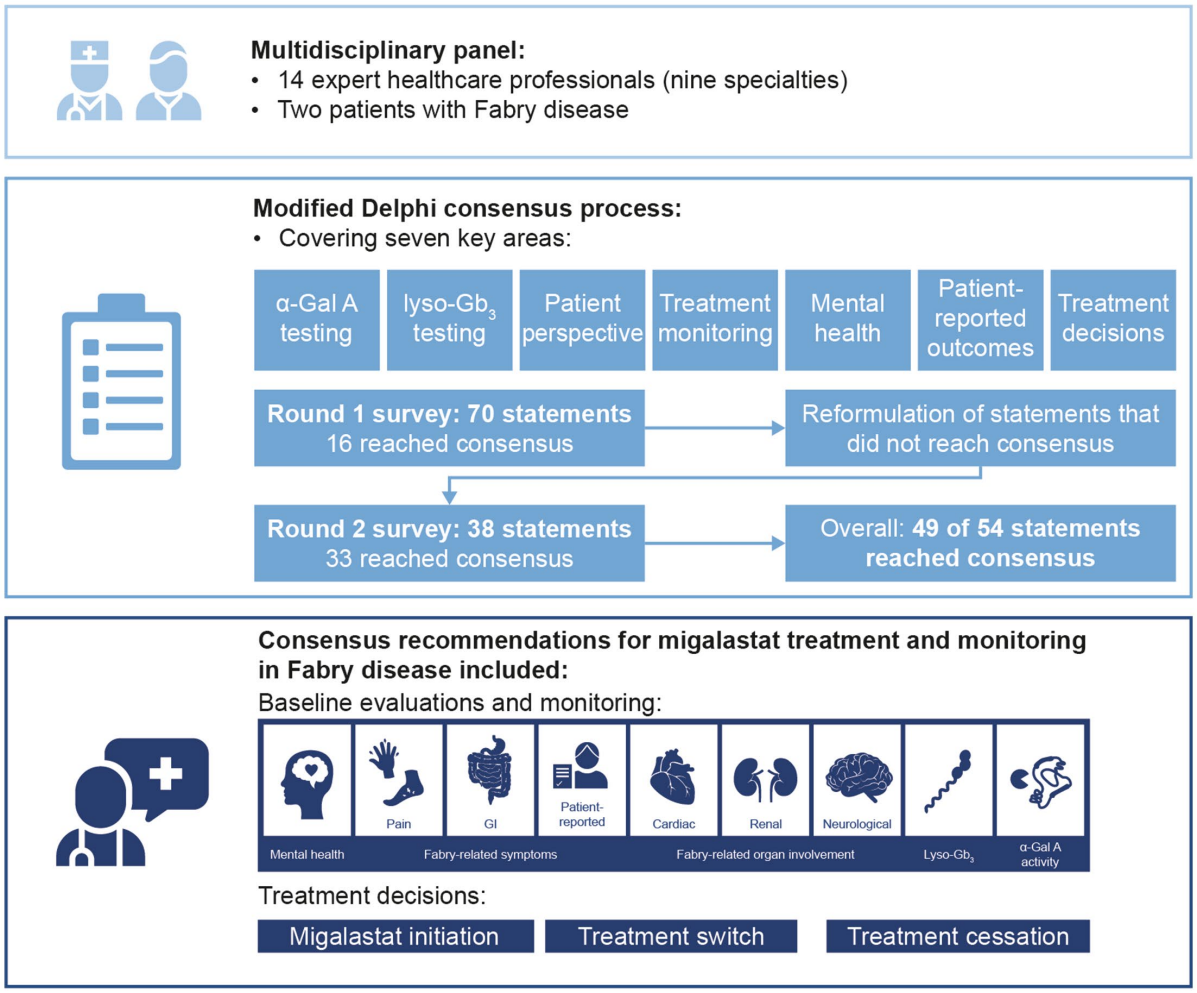

## 1. Introduction

Fabry disease is a progressive, multisystemic, X-linked disorder caused by variants in the *GLA* gene resulting in partial or absolute deficiency of α-galactosidase A (α-Gal A) activity (1–3). This leads to progressive intracellular accumulation of glycosphingolipids, primarily globotriaosylceramide (Gb_3_), and globotriaosylsphingosine (lyso-Gb_3_) (2, 3), causing multisystem cellular dysfunction, chronic inflammation, and fibrosis (4, 5), and ultimately resulting in irreversible tissue and organ damage (5–12).

Fabry disease is highly variable in terms of age of onset, symptom presentation, and organ involvement (2, 13, 14). Patient presentations may be broadly classified as an early-onset classical phenotype and a non-classic, late-onset phenotype (13, 14). Patients with the classic phenotype present with absent or very low α-Gal A activity, resulting in a severe disease course leading to peripheral manifestations of angiokeratoma, acroparesthesia, cornea verticillata and sweating abnormalities, as well as multiorgan failure and premature death if untreated (13). The late-onset phenotype encompasses patients with higher levels, although still deficient, of residual α-Gal A activity and disease predominantly of the heart. Notably, this is often without the peripheral manifestations of pain, eye changes, and sweating abnormalities (13, 15–18). Recent studies have demonstrated that the late-onset phenotype is more common than previously estimated (17, 19), with potential late-onset *GLA* variants found in over 88% of patients with Fabry disease identified by newborn screening across Taiwan, Italy, Japan, and New York (20–24). Systematic screening programs for Fabry disease predominantly identify male patients (17, 20–23, 25, 26), with enzyme-based screening approaches being less reliable for female patients who may present with normal plasma or leukocyte α-Gal A activity (19, 27).

Intravenous enzyme replacement therapy (ERT) with agalsidase beta (Fabrazyme®) is approved for patients with Fabry disease aged ≥2 years in the United States of America (USA) and ≥8 years in Europe (28, 29), and ERT with agalsidase alfa (Replagal®) is approved for use in patients with Fabry disease, with dosage recommendations for ages 7–65 years (30). An international panel of Fabry disease experts across seven subspecialties developed consensus recommendations for management and treatment of adults with Fabry disease receiving ERT which were published in 2018 (15). These recommendations highlighted the importance of monitoring both Fabry-related symptoms and organ involvement (15). Due to initial asymptomatic presentation of patients diagnosed through family screening, treatment is not always initiated immediately following diagnosis (31). Multidisciplinary monitoring can help to facilitate early treatment initiation, which is important to avoid irreversible tissue/organ damage (15, 31–33). Monitoring frequency can vary by phenotype (15), but one should always consider the whole clinical picture relevant to an individual. This includes key affected organs, i.e., cardiac, renal, and cerebrovascular, symptoms such as pain and gastrointestinal (GI) manifestations, and negative impacts on mental health and quality of life (QoL) (15, 34–37).

The oral chaperone therapy migalastat (Galafold^®^; Amicus Therapeutics, Philadelphia, PA, United States) is approved for use in patients with Fabry disease with amenable *GLA* mutations aged ≥16 years in Australia, ≥12 years in Europe, and ≥18 years in the United States and Canada (38–41). Amenability is defined using a good laboratory practice (GLP)-validated *in vitro* assay of human embryonic kidney (HEK) cells transfected with the *GLA* mutation and incubated with migalastat (42). Mutations are categorized as amenable if the transfected cells display a ≥1.2-fold increase above baseline and a ≥3% absolute increase in wild-type α-Gal A activity (42). As of May 2022, 1,386 variants were classified as amenable to migalastat (39); labeling may vary by region (38, 40) and this overall value is updated as new variants are identified and tested in the amenability assay.

Owing to the different mechanisms of action and approved indications for migalastat and ERT, they may require different monitoring and treatment guidelines. For example, α-Gal A activity has been hypothesized to provide an indication of the biological activity of migalastat (42–45), suggesting potential value as a pharmacodynamic response biomarker. Additionally, lyso-Gb_3_ may not correlate with clinical outcomes in patients treated with either migalastat or ERT (46, 47), although its use at diagnosis may aid in predicting disease progression (48–51). Consideration should also be given to defining disease progression, stability, and improvement in patients with Fabry disease, in order to aid treatment decisions in a landscape where there are now multiple options available. Consistency in the monitoring of patients on migalastat will help to evaluate treatment-specific outcomes (15).

We conducted a modified Delphi process (52) with a panel of expert physicians and patients with Fabry disease to determine consensus on treatment initiation and monitoring of patients receiving migalastat; the results and recommendations are reported here.

## 2. Methods

**Figure 1:**
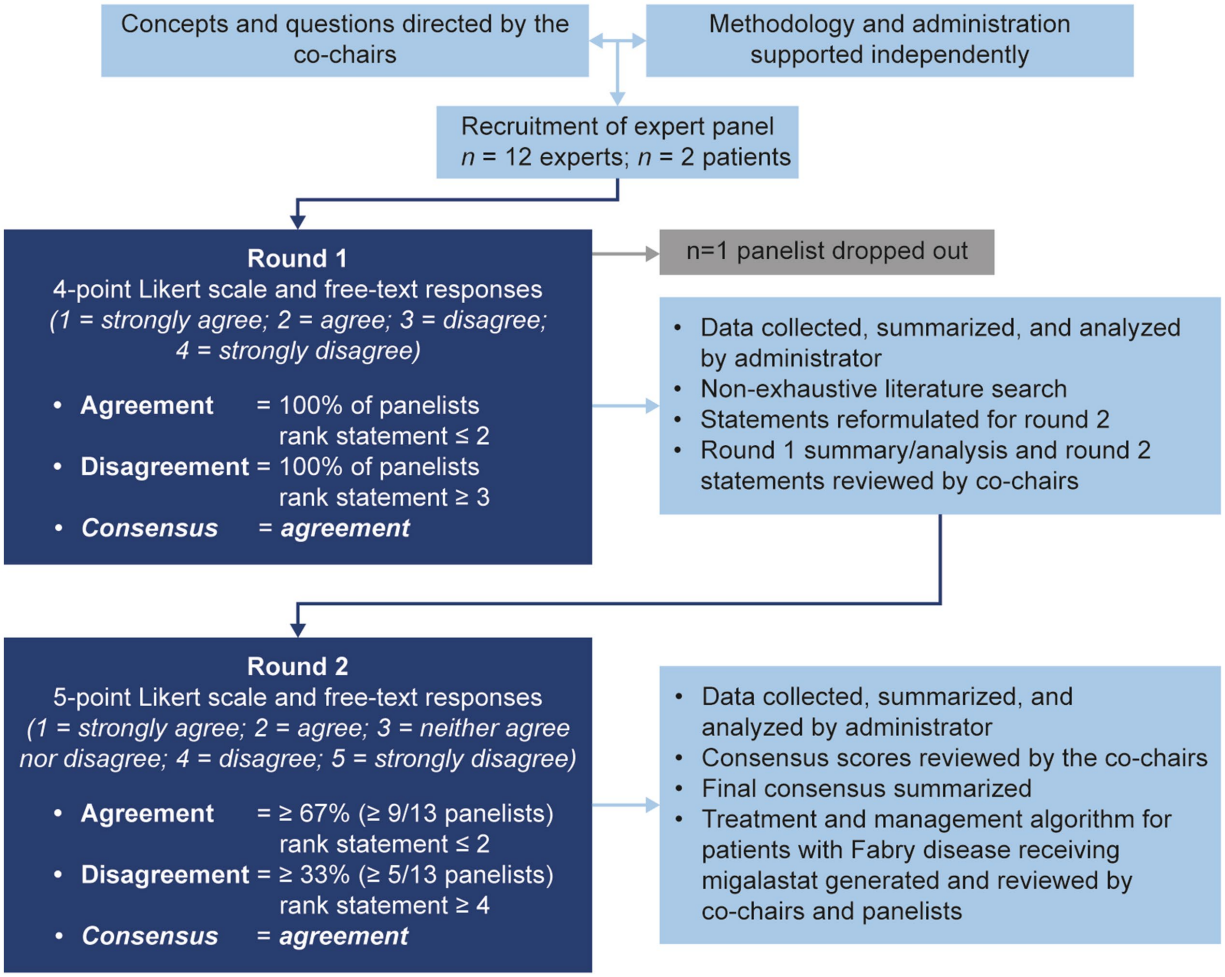
The modified Delphi methodology used in this study.

The modified Delphi process used in this study is summarized in Figure 1. A consultative survey-based technique was used to reach consensus on best practice for the management of Fabry disease with migalastat using a modified Delphi approach (52, 53).

### 2.1. Selection of co-chairs and expert panel

The co-chairs and expert panel were selected from participants of a series of three roundtable meetings (in-person and virtual) that occurred between September and December 2020. Meeting attendees included expert physicians and Fabry patient advocates. Meeting attendees were compensated for their participation in roundtable meetings; none of the authors were compensated for their input into the Delphi process or the publication. Expert physicians came from a wide range of Fabry-related medical disciplines, and all had experience in management of patients with Fabry disease on migalastat. The meetings aimed to identify key areas where updates to existing treatment and monitoring guidelines were required, specifically in relation to management of patients with Fabry disease on migalastat.

Three of the leading global experts in Fabry disease in attendance were invited, and subsequently agreed, to be co-chairs for this modified Delphi consensus study. Fourteen panelists agreed to participate in the consensus survey, including 12 expert panelists and two patient advocates, all of whom attended one or more of the meetings. One expert declined to participate after round 1 and was excluded from the analysis. Overall, the three co-chairs and 11 expert panelists represented 11 countries and eight specialties (Table 1).

**Table 1:** Expert physician author experience with Fabry disease and migalastat (*n* = 3 co-chairs; *n* = 11 expert panelists).

**Country**
Argentina	1
Brazil	1
Canada	1^*^
France	1
Italy	1
Norway	1
Portugal	1
Spain	1
Taiwan	2
United Kingdom	1^*^
United States	3^*^
**Specialty**^†^
Cardiology	1
Genetics	5^*^
Hematology	1^*^
Lysosomal diseases	1
Metabolism	2
Nephrology	4^*^
Neurology	1
Pediatrics	3^*^
Internal medicine	2
**Duration of Fabry disease clinical experience, years**
Mean (SD)	20 (6)
0–10	0
11–20	7
21–30	7
>30	0
**Experience with migalastat (research, managing patients), years**	
Mean (SD)	7 (4)
0–5	8
5–10	3
>10	3
**Number of patients with Fabry disease managed**
Mean (SD)	123 (121)
1–10	0
11–50	4
51–100	4
>100	6
**Summary of patients managed, mean (range)**
Male	55 (12–200)
Female	64 (0–300)
Classic	69 (10–250)
Non-classic	50 (2–250)

^*^Indicates *n* = 1 co-chair; ^†^Some experts have multiple specialties.

SD, standard deviation.

The consensus committee convened to agree on the objectives of the study and provide guidance on the development of Delphi statements. Before the meeting, a non-exhaustive literature search was conducted (see Supplementary Appendix A.1) to identify guidelines or management recommendations for patients with Fabry disease receiving migalastat, with results presented to the committee during the meeting. Overall, seven sources were identified that provided country-specific recommendations for migalastat (from Spain, Germany, United Kingdom, Italy, and Canada) (54–60). No international guidelines for migalastat were available. During the meeting, the committee agreed that the consensus process should build on available recommendations for Fabry disease and migalastat by focusing on key decision and communication points within the journey of a patient with Fabry disease receiving migalastat: diagnosis, baseline assessments, treatment decisions, and continual monitoring. During the meeting, the following key areas where a consensus opinion would be beneficial were agreed: the utility of α-Gal A and lyso-Gb_3_ monitoring, the importance of the patient perspective, treatment monitoring [including mental health and patient-reported outcomes (PROs)], and criteria that may inform treatment decisions.

### 2.2. Modified Delphi process

All stages of the Delphi process were overseen by the co-chairs and conducted by an independent third-party administrator (Comradis, London, United Kingdom). Seventy Delphi statements were developed by the administrator to focus the consensus process on the key areas identified in the consensus committee meeting. Results from the initial non-exhaustive literature search (Supplementary Appendix A.1) along with expert opinion from discussions during the meeting were used to inform the development of the statements.

Panelist responses were gathered by the administrator via an online survey platform for round 1 and a questionnaire document for round 2. Responses were anonymized before sharing with the co-chairs. Circulation of the questionnaires, collection of responses, and processing of responses was conducted between March and July 2022.

In round 1, panelists were asked to complete an anonymous questionnaire with 70 statements (Supplementary Appendix B). A summary of the key decision and communication points identified during the committee meeting was provided to all panelists with the round 1 statements. Statements were ranked using a four-point Likert scale (1 = strongly agree, 2 = agree, 3 = disagree, and 4 = strongly disagree) and panelists were asked to provide rationale/supporting evidence or reasoning for disagreement in a free-text response. Agreement was defined as 100% of responses ranked ≤2; statements that reached agreement in round 1 were determined to have reached consensus. The administrator collated all round 1 responses and performed a non-exhaustive literature search guided by panelists’ Likert scale and free-text responses (Supplementary Appendix A.2). Statements that did not achieve consensus were reformulated and presented with supporting literature from the two non-exhaustive literature searches (Supplementary Appendix A) for round 2. Statements that achieved consensus in round 1 (*n* = 16) were not reformulated for round 2. The round 1 statements, a selection of representative anonymized panelists’ responses from round 1, the percentage agreement or disagreement from round 1, a summary of relevant literature from a non-systematic literature search, and the statements for round 2 (*n* = 38) were presented to the co-chairs for review, before being presented to the panelists (Supplementary Appendix A).

In round 2, panelists ranked statements on a five-point Likert scale (1 = strongly agree, 2 = agree, 3 = neither agree nor disagree, 4 = disagree, and 5 = strongly disagree). The five-point scale was selected for round 2 because free-text responses from round 1 strongly indicated that a neutral option would be beneficial for areas outside of panelists’ expertise. Agreement was defined *a priori* as ≥67% (two-thirds, or ≥ 9/13 panelists) of responses ranked ≤2. Disagreement was defined *a priori* as ≥33% (one-third, or ≥ 5/13 panelists) of responses ranked ≥4. Statements that reached agreement in round 2 were determined to have reached consensus. Statement Likert scale ranks were compiled by the administrator and reviewed by the co-chairs, as in previous modified Delphi methods (31, 61).

After reviewing panelists’ anonymized free-text responses to the statements that did not reach consensus criteria, the co-chairs agreed that there was such a large difference of opinion between panelists (that was not resolved with reformulation in round 2) that further reformulation of the statements in a third round would not be more likely to reach consensus. Statements that reached consensus were used to generate recommendations for monitoring and treatment decisions in patients with Fabry disease. Statements that did not reach consensus are addressed in the discussion.

### 2.3. Statistical analyses

All data are reported descriptively because of the exploratory nature of the Delphi consensus process. To standardize responses between rounds 1 and 2, Likert scale rankings were assigned a numerical score between −2 and 2 (strongly disagree = −2; disagree = −1; neither agree nor disagree = 0; agree = 1; strongly agree = 2); as per a previously reported Delphi study (53), mean consensus scores were then calculated for each statement to give an indication of the relative degree of agreement/disagreement across statements. Results are presented as mean consensus score, along with the number of panelists with an agreement score (≥ 1) or disagreement score (≤ −1) and whether the statement reached consensus according to the *a priori*-defined criteria (see Figure 1). A mean consensus score of 0.1 is the lowest possible score where agreement (as per our *a priori* definition of ≥67% of responses ranked ≤2) can be reached. A mean consensus score of 1.0 is the lowest possible score where all panelists responded with agree or strongly agree. Moderate consensus was therefore considered to be a mean consensus score of 0.1–1.0; strong consensus was considered to be a score of 1.0–2.0.

## 3. Results

Overall, 16/70 round 1 statements and 33/38 round 2 statements reached consensus across four key areas: α-Gal A measurements in patients receiving migalastat, lyso-Gb_3_, treatment monitoring (including PROs, the patient perspective, and mental health), and criteria for making treatment switch/stop decisions. Considerations for migalastat made up 59% of the statements; given the importance of the patient experience and multisystemic involvement of Fabry disease, the remainder of the statements considered monitoring and treatment decisions in the context of these topics.

Of the nine statements regarding α-Gal A measurement in patients receiving migalastat, six reached consensus, two reached disagreement, and one did not reach either criterion (Table 2). Of the 14 statements regarding lyso-Gb_3_ measurement in patients with Fabry disease, 12 reached consensus (three at round 1; nine at round 2) and two did not reach either criterion by round 2 (Table 3). All 10 statements regarding monitoring assessments in patients with Fabry disease reached consensus (two at round 1; eight at round 2; Table 4). All 21 statements regarding treatment decisions in patients with Fabry disease reached consensus (Table 5). All three statements regarding patient involvement and 8/13 statements regarding treatment initiation reached consensus in round 1. Recommendations for monitoring and treatment decisions in patients with Fabry disease based on the consensus results are presented in Table 6.

**Table 2:** Results of the round 1 and 2 modified Delphi consensus process relating to α-Gal A measurement in patients receiving migalastat.

ID	Statement	Rank,^*^ *n*			Verdict	Mean consensus score^†^
		Agreement	Neutral	Disagreement		
1A	Migalastat should be continued in female patients with amenable mutations who are showing a stabilization or improvement in Fabry-related symptoms, regardless of the change in α-Gal A activity	10	2	1	Consensus (agreement)	1.2
1B	Measurement of α-Gal A activity in female patients should not be used to make decisions about whether to continue migalastat treatment	12	0	1	Consensus (agreement)	1.1
1C	In male patients receiving migalastat, α-Gal A enzyme activity in leukocytes should be measured before initiation of migalastat and as part of routine follow-up	9	3	1	Consensus (agreement)	1.0
1D	Migalastat should be continued in male patients with amenable mutations who are showing a stabilization or improvement in Fabry-related symptoms, regardless of the change in α-Gal A activity	10	1	2	Consensus (agreement)	1.0
1E	Migalastat should be continued in both male and female patients with stable or improved organ function, regardless of any change in the α-Gal A activity	10	0	3	Consensus (agreement)	0.8
1F	α-Gal A activity in leukocytes should be routinely measured from migalastat initiation to inform an assessment of the biological activity of migalastat	9	1	3	Consensus (agreement)	0.6
1G	In male patients receiving migalastat, an increase in α-Gal A activity to 10–20% of normal levels indicates biological activity of migalastat and supports a clinical decision to continue treatment	8	2	3	Criteria not reached	0.5
1H	An increase in α-Gal A activity by 20–30% from baseline indicates biological activity of migalastat and supports a clinical decision to continue treatment	5	3	5	Disagreement	0.1
1I	In female patients receiving migalastat, α-Gal A enzyme activity in leukocytes should be measured before initiation of migalastat and as part of routine follow-up	1	6	6	Disagreement	−0.4

* The number of panelists with a Likert scale rank of agreement (“agree” or “strongly agree”), neutral (“neither agree nor disagree”), or disagreement (“disagree” or “strongly disagree”) for each statement is presented;

† Strongest agreement = 2, strongest disagreement = –2.

α-Gal A, α-galactosidase A.

**Table 3:** Results of the round 1 and 2 modified Delphi consensus process relating to lyso-Gb_3_ measurement in patients with Fabry disease.

ID	Statement	Rank,^*^ *n*			Verdict	Mean consensus score^†^
		Agreement	Neutral	Disagreement		
2A^‡^	Lyso-Gb_3_ analysis should be performed in all patients at diagnosis	13	0	0	Consensus (agreement)	1.5
2B^‡^	Following any increase in lyso-Gb_3_ from baseline, where there has been no change in treatment, patients should be asked about their adherence to migalastat	13	0	0	Consensus (agreement)	1.5
2C	Adherence and compliance with migalastat should be discussed with the patient in a systematic way, reviewing the patient’s current dosing and posology	13	0	0	Consensus (agreement)	1.5
2D	Current migalastat treatment should be continued in any patient showing stable or improved organ function, if lyso-Gb_3_ is stable or declining	12	1	0	Consensus (agreement)	1.3
2E	In ERT-experienced male patients switched to migalastat, stable (or declining) lyso-Gb_3_ levels after 12 months of migalastat treatment, relative to lyso-Gb_3_ levels while receiving ERT, indicate biological activity of migalastat and support a clinical decision to continue treatment	12	0	1	Consensus (agreement)	1.1
2F	Current migalastat treatment should be continued in any patient showing a symptomatic response (response in symptoms), if lyso-Gb_3_ is stable or declining	12	1	0	Consensus (agreement)	1.1
2G	Lyso-Gb_3_ analysis should be performed every 6 months–1 year in patients receiving treatment with migalastat	11	1	1	Consensus (agreement)	1
2H	In treatment-naïve male patients, a decrease in lyso-Gb_3_ from pre-treatment baseline within ≤12 months after migalastat initiation that is sustained indicates biological activity of migalastat	10	3	0	Consensus (agreement)	1
2I	In ERT-experienced female patients switched to migalastat, stable (or declining) lyso-Gb_3_ levels after 12 months of migalastat treatment, relative to lyso-Gb_3_ levels while receiving ERT, indicate biological activity of migalastat and support a clinical decision to continue treatment	11	0	2	Consensus (agreement)	0.9
2J	Lyso-Gb_3_ analysis should be performed every 6 months–1 year in patients receiving treatment with ERT	10	1	2	Consensus (agreement)	0.8
2K	In treatment-naïve female patients, any decrease in lyso-Gb_3_ from pre-treatment baseline within ≤12 months after migalastat initiation indicates biological activity of migalastat	9	4	0	Consensus (agreement)	0.8
2L	In treatment-naïve female patients, failure to reduce lyso-Gb_3_ from pre-treatment baseline within ≤12 months of migalastat treatment is an indication to consider changing treatment	9	2	2	Consensus (agreement)	0.6
2M	In treatment-naïve male patients, failure to reduce lyso-Gb_3_ from pre-treatment baseline within ≤12 months of migalastat treatment is an indication to consider changing treatment	8	2	3	Criteria not reached	0.5
2N	Lyso-Gb_3_ is a reliable pharmacodynamic biomarker for monitoring treatment response in patients receiving migalastat	5	5	3	Criteria not reached	0.3

^*^The number of panelists with a Likert scale rank of agreement (“agree” or “strongly agree”), neutral (“neither agree nor disagree”), or disagreement (“disagree” or “strongly disagree”) for each statement are presented; ^†^Strongest agreement = 2, strongest disagreement = –2; ^‡^Indicates statement reached consensus in round 1.

ERT, enzyme replacement therapy; lyso-Gb_3_, globotriaosylsphingosine.

**Table 4:** Results of the round 1 and 2 modified Delphi consensus process relating to treatment monitoring.

ID	Statement	Rank,^*^ *n*			Verdict	Mean consensus score^†^
		Agreement	Neutral	Disagreement		
	**Mental health and PROs**					
3A^‡^	All patients with Fabry disease should be evaluated for pain and GI symptoms at baseline (i.e., prior to treatment initiation)	13	0	0	Consensus (agreement)	1.6
3B^‡^	All patients with Fabry disease should be evaluated for mental health by a healthcare professional, using a validated screening tool	13	0	0	Consensus (agreement)	1.5
3C	All patients with Fabry disease should have a mental health assessment at baseline and at 12-month intervals by a healthcare professional, using a validated screening tool	13	0	0	Consensus (agreement)	1.2
3D	All patients with Fabry disease should have interval evaluations with PROs (e.g., EQ-5D, SF-36) every 6–12 months, depending on disease severity, patient needs, and usual frequency of specialist appointments	13	0	0	Consensus (agreement)	1.2
3E	As part of routine care, all patients should be referred to an appropriate mental healthcare professional (e.g., genetic counselor, licensed therapist, social worker, and psychologist)	11	1	1	Consensus (agreement)	0.9
	**Organ function**					
4A	All patients should undergo an evaluation of Fabry-related symptoms at baseline and every 6–12 months thereafter, depending on disease severity	13	0	0	Consensus (agreement)	1.5
4B	All patients with Fabry disease should have an evaluation of renal and cardiac function at baseline and every 6–12 months thereafter, depending on disease severity, clinical presentation, and patient needs	12	1	0	Consensus (agreement)	1.4
4C	For any patient presenting with severe or rapidly declining symptoms and/or organ involvement from baseline on two consecutive readings at least 6 months apart, more frequent follow-up evaluations (approximately every 3 months) should be considered	12	1	0	Consensus (agreement)	1.4
4D	All patients with Fabry disease should have an evaluation of neurological function at baseline and every ≤3 years thereafter, depending on disease severity and clinical presentation of neurological symptoms	12	1	0	Consensus (agreement)	1.2
4E	A one-time follow-up evaluation at 3 months’ post-treatment initiation or switch should be considered before deciding on the frequency of subsequent follow-up evaluations	10	1	2	Consensus (agreement)	0.8

^*^The number of panelists with a Likert scale rank of agreement (“agree” or “strongly agree”), neutral (“neither agree nor disagree”), or disagreement (“disagree” or “strongly disagree”) for each statement are presented; ^†^Strongest agreement = 2, strongest disagreement = –2; ^‡^Indicates statement reached consensus in round 1.

EQ-5D, European quality of life-5 dimensions; GI, gastrointestinal; PRO, patient-reported outcome; SF-36, 36-item short form health survey.

**Table 5:** Results of the round 1 and 2 modified Delphi consensus process relating to treatment decisions.

ID	Statement	Rank,^*^ *n*			Verdict	Mean consensus score^†^
		Agreement	Neutral	Disagreement		
	**Patient involvement**					
5A^‡^	Patients should be given an overview of all suitable available therapies	13	0	0	Consensus (agreement)	1.8
5B^‡^	Patients should be encouraged to be involved in shared decision-making when starting, changing, or stopping therapy	13	0	0	Consensus (agreement)	1.8
5C^‡^	Patient choice should be taken into consideration in regard to any decision to start therapy, change therapy, or stop therapy	13	0	0	Consensus (agreement)	1.6
	**Treatment initiation**					
6A^‡^	Male patients may be started on migalastat if they have evidence of Fabry-related symptoms and an amenable mutation	13	0	0	Consensus (agreement)	1.6
6B^‡^	Female patients may be started on migalastat if they have evidence of Fabry-related symptoms and an amenable mutation	13	0	0	Consensus (agreement)	1.6
6C^‡^	Female patients with classic mutations that are amenable to migalastat should commence treatment if they have evidence of organ involvement	13	0	0	Consensus (agreement)	1.6
6D	Male patients may be started on migalastat if they have evidence of Fabry-related organ involvement and an amenable mutation	13	0	0	Consensus (agreement)	1.5
6E	Female patients may be started on migalastat if they have evidence of Fabry-related organ involvement and an amenable mutation	13	0	0	Consensus (agreement)	1.5
6F^‡^	Patients with classic or late-onset Fabry disease may both be started on migalastat if they have evidence of Fabry-related symptoms and an amenable mutation	13	0	0	Consensus (agreement)	1.5
6G^‡^	Male and female patients with late-onset mutations that are amenable to migalastat should commence treatment if they have evidence of organ involvement	13	0	0	Consensus (agreement)	1.5
6H^‡^	Patients with classic or late-onset Fabry disease may both be started on migalastat if they have evidence of organ involvement and an amenable mutation	13	0	0	Consensus (agreement)	1.4
6I^‡^	Female patients may be started on migalastat in the presence of a classic Fabry mutation, amenable to migalastat, if they have at least one Fabry-related symptom	13	0	0	Consensus (agreement)	1.4
6J^‡^	Migalastat and enzyme replacement therapy should follow the same guidelines and recommendations when it comes treatment cessation	13	0	0	Consensus (agreement)	1.3
6K	Family history should be considered when deciding whether to initiate treatment, but is not the only factor for this decision	13	0	0	Consensus (agreement)	1.3
6L	Male patients may be started on migalastat in the presence of a classic Fabry mutation, amenable to migalastat, even in the absence of organ involvement	12	1	0	Consensus (agreement)	1.3
6M	There should be a single set of treatment guidelines and recommendations to follow for initiation and cessation of both treatments for Fabry disease (migalastat and enzyme replacement therapy), including criteria such as amenability and enzyme activity measurements	10	1	2	Consensus (agreement)	0.8
	**Treatment switch/stop**					
7A	When considering whether to switch or stop treatment in patients with Fabry disease, treatment compliance, patient-reported outcomes, and patient choice should be taken into account	13	0	0	Consensus (agreement)	1.5
7B	Migalastat treatment should be continued in patients with stable or improving Fabry-related symptoms, even if they show no improvement in organ function from baseline	12	1	0	Consensus (agreement)	1.2
7C	Deterioration of renal, neurological, or cardiac organ function (or architecture) from baseline on two consecutive readings at least 6 months apart is an indication to consider switching ERT or migalastat treatment due to lack of efficacy	11	1	1	Consensus (agreement)	1
7D	Deterioration of symptoms alone from baseline on two consecutive readings at least 6 months apart is not an indication to consider stopping or switching treatment; organ involvement, patient-reported outcomes, and patient choice should also be considered	11	1	1	Consensus (agreement)	0.9
7E	Patients with stabilization or improvement from baseline in ≥1 organ should consider remaining on treatment, even if function of another organ deteriorates	11	1	1	Consensus (agreement)	0.9

^*^The number of panelists with a Likert scale rank of agreement (“agree” or “strongly agree”), neutral (“neither agree nor disagree”), or disagreement (“disagree” or “strongly disagree”) for each statement are presented; ^†^Strongest agreement = 2, strongest disagreement = –2; ^‡^Indicates statement reached consensus in round 1.

ERT, enzyme replacement therapy.

**Table 6:** Recommendations for α-Gal A measurement and lyso-Gb_3_, in patients with Fabry disease.

α-Gal A measurements in patients receiving migalastat	Lyso-Gb_3_ measurements
**No consensus was reached regarding a threshold percentage increase in α-Gal A activity from baseline or normal levels that would indicate biological activity of migalastat** ***(statements 1G and 1H; no consensus [0.5 and 0.1, respectively])***; **therefore, the utility of α-Gal A as a pharmacodynamic treatment response biomarker for migalastat remains unclear.** α-Gal A activity in leukocytes should be measured before migalastat initiation and during routine follow-up to inform assessment of the biological activity of migalastat (*statement 1F*; *moderate consensus [0.6]*). In female patients receiving migalastat, α-Gal A activity should not be used to make decisions about whether to continue migalastat treatment (*statement 1B; strong consensus [1.1]*). Migalastat should be continued in female patients showing stabilization or improvement in Fabry-related symptoms and/or organ involvement, regardless of the change in α-Gal A activity (*statement 1A; strong consensus [1.2]*). In male patients receiving migalastat, α-Gal A activity should be measured as part of routine follow-up (*statement 1C; moderate consensus [1.0]*). Migalastat should be continued in male patients showing stabilization or improvement in Fabry-related symptoms, regardless of the change in α-Gal A activity (*statement 1D; moderate consensus [1.0]*). Migalastat should be continued in both male and female patients with stable or improved organ function, regardless of any change in α-Gal A activity (*statement 1E; moderate consensus [0.8]*).	**No consensus was reached regarding the reliability of lyso-Gb_3_ as a pharmacodynamic biomarker for monitoring treatment response in patients with Fabry disease receiving migalastat** ***(statement 2N; no consensus [0.3])***. **This should be considered when evaluating all other lyso-Gb_3_-related recommendations**. Lyso-Gb_3_ analysis should be performed in all patients at diagnosis (*statement 2A; strong consensus [1.5]*), and every 6 months–1 year in patients receiving either ERT (*statement 2J; moderate consensus [0.8]*) or migalastat (*statement 2G; moderate consensus [1.0]*). In both male and female treatment-naïve patients, a decrease in lyso-Gb_3_ from BL within ≤12 months after migalastat initiation indicates biological activity of migalastat (*statements 2H and 2K; moderate consensus [1.0 and 0.8, respectively]*). In ERT-experienced male (*statement 2E; strong consensus [1.1]*) or female (*statement 2I; moderate consensus [0.9]*) patients switched to migalastat, stable or declining lyso-Gb_3_ levels after 12 months of migalastat treatment relative to during ERT treatment indicate biological activity of migalastat and support a clinical decision to continue treatment. Migalastat treatment should be continued in any patient showing a symptomatic response and/or stable or improved organ function, if lyso-Gb_3_ is stable or declining (*statements 2F and 2D; strong consensus [1.1 and 1.3, respectively]*). Following any increase in lyso-Gb_3_ from BL, where there has been no change in treatment, patients should be asked about their adherence to migalastat (*statement 2B; strong consensus [1.5]*). Treatment adherence with migalastat should be discussed in a systematic way, reviewing the patient’s current dosing and posology (*statement 2C; strong consensus [1.5]*).

Food and caffeine should not be consumed at least 2 h prior to and 2 h after taking migalastat to give a minimum 4 h fast (38, 39).

α-Gal A, α-galactosidase A; BL, baseline; ERT, enzyme replacement therapy; and lyso-Gb_3_, globotriaosylsphingosine.

## 4. Discussion

### 4.1. α-Gal A measurements in patients receiving migalastat

Recommendations based on consensus results for α-Gal A are presented in Table 6. The panel reached consensus on the recommendation to monitor α-Gal A activity in patients receiving migalastat (statements 1C and 1F); however, α-Gal A activity should not inform clinical decision-making (statements 1A, 1B, 1D, and 1E). Decisions on whether to continue migalastat in both male and female patients should be made regardless of changes in α-Gal A activity; such decisions should utilize a holistic assessment, considering clinical factors such as Fabry-related symptoms and/or organ involvement. The utility of α-Gal A as a pharmacodynamic response biomarker remains unclear. The routine, systematic measurement of α-Gal A activity in leukocytes in both male and female patients on migalastat (statements 1C and 1F) could inform investigators about the validity and relevance of α-Gal A as a potential biomarker to assess treatment response. Collection and entry of these data into a registry (such as the followME Fabry Pathfinders registry) could in part facilitate this.

Notably, consensus was not reached regarding an *in vivo* threshold increase in α-Gal A activity from baseline or normal levels that would indicate biological activity of migalastat. The threshold increase in the *in vitro* GLP-validated amenability assay is 20% from baseline activity (42); however, how this translates into an *in vivo* threshold increase is unknown (62). Specific protocols considering α-Gal A pharmacokinetic (PK) and pharmacodynamic parameters (e.g., time from migalastat dose and time from blood draw to enzyme activity measurement) are required to standardize α-Gal A measurement across centers so that individual patient results can be compared. Additionally, whether there is a correlation between α-Gal A in leukocytes and podocytes/cardiomyocytes is unknown. Potential research avenues to investigate the identified data gaps for α-Gal A are described in Table 7.

**Table 7:** Recommendations for further research based on the modified Delphi consensus results (consensus scores and free-text responses).

α-Gal A^*^ PK/PD studies for α-Gal A post-migalastat and post-blood draw. α-Gal A levels in different cell types (leukocytes, podocytes, and cardiomyocytes) throughout disease progression and their relationship to plasma α-Gal A and lyso-Gb_3_^†^. Relationship of α-Gal A to clinical outcomes in patients receiving migalastat [analysis by phenotype (classic/late-onset) and sex]. Data entry into registries to enable analysis of larger cohorts over time.
**Lyso-Gb**_3_^*^ Lyso-Gb_3_ and clinical outcomes in patients receiving migalastat or ERT^†^. Data entry into registries to enable analysis of larger cohorts over time. Molecular studies evaluating the mechanism by which lyso-Gb_3_ is related to disease progression. Collect “baseline” and post-treatment (re)initiation lyso-Gb_3_ values from patients who experience a ≥ 1-month treatment interruption.
**Treatment guidelines** Single set for ERT and migalastat, including similarities and differences in initiation and monitoring.

^*^α-Gal A and lyso-Gb_3_ testing can be accessed through multiple laboratories (63). Availability and standardization of testing for these parameters is key; ^†^This could be assessed using a prospective study of α-Gal A and lyso-Gb_3_ in patients with amenable mutations receiving migalastat over a period of ≥2 years and including patients across all phenotypes over a wide age range.

α-Gal A, α-galactosidase A; ERT, enzyme replacement therapy; lyso-Gb_3_, globotriaosylsphingosine; PD, pharmacodynamic; and PK, pharmacokinetic.

Additionally, there was disagreement regarding the utility of α-Gal A measurement at follow-up in female patients receiving migalastat (statement 1I; no consensus [−0.4]) given that 40% of female patients present with normal leukocyte α-Gal A activity at baseline (64, 65). Panelists noted that monitoring of α-Gal A in female patients with Fabry disease receiving migalastat would be useful for research purposes to better characterize its change over time. In male or female patients in whom α-Gal A is abnormal at baseline, we might expect improvement over time.

### 4.2. Lyso-Gb_3_

Recommendations based on consensus results for lyso-Gb_3_ are presented in Table 6.

Our consensus results support a lack of agreement within the field regarding the utility of lyso-Gb_3_ as a pharmacodynamic biomarker for monitoring treatment response in patients with Fabry disease. While it was recommended that lyso-Gb_3_ analysis should be carried out at diagnosis and follow-up, no consensus was reached regarding the reliability of lyso-Gb_3_ as a pharmacodynamic biomarker for monitoring treatment response in patients with Fabry disease receiving migalastat.

In the PREDICT-FD modified Delphi consensus initiative, there was similarly no consensus reached on the use of lyso-Gb_3_ as an indicator for treatment initiation (31). Within the literature, there is also conflicting evidence regarding the utility of lyso-Gb_3_ as a biomarker to monitor treatment response (13, 46, 47, 49). Although plasma lyso-Gb_3_ has been shown to decrease or stabilize in patients receiving treatment with ERT (47, 66, 67) and migalastat (45, 68), several studies demonstrated that neither lyso-Gb_3_ concentration nor rate of change predicts the risk of Fabry-associated clinical events in either ERT-or migalastat-treated patients (46, 47). Additionally, the exact mechanism by which substrate accumulation acts in Fabry disease is not completely understood (5, 46). Continued lyso-Gb_3_ measurement at follow-up in patients receiving migalastat will provide further evidence on the extent of the relationship between lyso-Gb_3_ and clinical outcomes in patients receiving treatment for Fabry disease, which may differ depending on sex and phenotype severity.

Similar to α-Gal A, some patients may present with lyso-Gb_3_ within the normal range at diagnosis, particularly patients with late-onset phenotypes and females; lyso-Gb_3_ may therefore be more useful as a biomarker in patients in whom it is clearly elevated at baseline, while further research is needed in patients with late-onset phenotypes and females. Additionally, some female patients present with elevated lyso-Gb_3_ but normal α-Gal A activity at diagnosis (48, 51, 69); the importance of each parameter for treatment monitoring may differ depending on its baseline value.

Responses to migalastat in treatment-naïve compared with treatment-experienced patients may be different; in treatment-naïve patients, a decrease in lyso-Gb_3_ and improvement in symptoms might be expected, while stability may be acceptable in treatment-experienced patients. In this consensus process it was unclear whether a change in treatment should be considered in treatment-naïve patients in whom there is no decrease in lyso-Gb_3_ within 12 months of migalastat initiation. Although failure to reduce lyso-Gb_3_ from baseline within ≤12 months of migalastat initiation in treatment-naïve female patients was regarded as an indication to consider changing treatment (statement 2L; moderate consensus [0.6]), panelists noted that this may not apply to all female patients; a reduction in lyso-Gb_3_ is not a therapeutic goal in female patients with normal lyso-Gb_3_ at baseline. No consensus was reached regarding failure to reduce lyso-Gb_3_ from pre-treatment baseline within ≤12 months of migalastat treatment being an indication to consider changing treatment in treatment-naïve male patients (statement 2M, no consensus [0.5]). Although reduction in lyso-Gb_3_ has been regarded as a treatment goal in males with classical Fabry disease, there is phenotype heterogeneity within males and therefore lyso-Gb_3_ should be considered in the context of the clinical picture of the patient.

The discrepancy between the consensus results on the utility of lyso-Gb_3_ to guide treatment decisions in treatment-naïve female and male patients receiving migalastat is driven by a single survey response, highlighting that there is not strong agreement in this area and that further research is required. In particular, the timeframe for potential changes in lyso-Gb_3_ may be considered; 12 months may not be enough time to demonstrate a decrease in lyso-Gb_3_.

Our recommendations note that if an increase in lyso-Gb_3_ from baseline is observed, treatment adherence with migalastat should be discussed (Table 6); however, this consensus initiative did not consider further treatment recommendations in this case. Further research is required to assess the utility of a marked increase in lyso-Gb_3_ as a biomarker to inform treatment decisions. Recommendations for treatment decisions are addressed in more detail in Section 3.5, highlighting that a holistic view of the patient must be considered.

### 4.3. Recommended monitoring assessments

Recommendations regarding organ involvement and Fabry-related symptoms (Table 8) aligned with the more extensive monitoring assessment recommendations described by Ortiz et al. (15). Strong consensus was reached regarding the need for all patients with Fabry disease to be regularly evaluated for mental health, PROs, pain, and GI symptoms. These evaluations can identify early symptoms of Fabry disease and facilitate timely treatment decisions, if appropriate. The recommendation for mental health monitoring supports an abundance of literature emphasizing the high prevalence of psychiatric disorders such as depression, anxiety, panic attacks, and social-adaptive dysfunction (15, 34, 35, 70). Follow-up frequency of each monitoring assessment should depend on the presentation of the patient, including clinical parameters and PROs. An appointment 3 months after any treatment initiation or switch should be considered to assess adverse events, treatment adherence, and doubts.

**Table 8:** Recommendations for monitoring and treatment decisions in patients with Fabry disease.

Monitoring assessments	Treatment decisions
**The following recommendations consider monitoring of Fabry-related symptoms and organ involvement in patients with Fabry disease. Recommendations regarding α-Gal A and lyso-Gb_3_ monitoring should also be considered.** All patients with Fabry disease should have a mental health assessment at BL and every 12 months thereafter conducted by an HCP, using a validated screening tool (*statements 3B and 3C; strong consensus [1.5 and 1.2, respectively]*). After the evaluation, as part of routine care, patients should be referred to an appropriate HCP, who can provide guidance/treatment for any identified issues (e.g., genetic counselor, licensed therapist, social worker, psychologist) (*statement 3E; moderate consensus [0.9]*). All patients with Fabry disease should be evaluated for pain and GI symptoms at BL prior to treatment initiation (*statement 3A; strong consensus [1.6]*). All patients with Fabry disease should have evaluations with PROs (e.g., EQ-5D, SF-36) every 6–12 months, depending on disease severity, patient needs, and the usual frequency of specialist appointments (*statement 3D; strong consensus [1.2]*). All patients with Fabry disease should have an evaluation of renal function, cardiac function (*statement 4B; strong consensus [1.4]*), and Fabry-related symptoms (*statement 4A; strong consensus [1.5]*) at BL and every 6–12 months thereafter, and neurological function at BL and every ≤3 years thereafter (*statement 4D; strong consensus [1.2]*). Frequency of follow-up evaluations should be decided based on disease severity, clinical presentation, and patient needs (*statements 4B and 4A*). For any patient presenting with severe or rapidly declining symptoms and/or organ involvement from BL on two consecutive readings at least 6 months apart, more frequent follow-up evaluations (approximately every 3 months) should be considered (*statement 4C; strong consensus [1.4]*). A one-time follow-up evaluation 3 months post-initiation or treatment switch should be considered before deciding on the frequency of subsequent follow-up evaluations (*statement 4E; moderate consensus [0.8]*).	Patients should be given an overview of all suitable available therapies (*statement 5A; strong consensus [1.8]*). Patients should be encouraged to be involved in treatment decisions, and their choice taken into consideration for any decision to start, change, or stop treatment (*statements 5B and 5C; strong consensus [1.8 and 1.6, respectively]*). There should be a single set of guidelines to follow for treatment initiation (*statement 6M; moderate consensus* *[0.8])* and cessation (*statement 6J; strong consensus [1.3]*) of both migalastat and ERT, capturing the similarities and differences between criteria for these treatments (e.g., approved indication and the requirement for amenability to migalastat). **Treatment initiation (in eligible patients according to licensed indications):** Family history (e.g., history of organ involvement, symptoms or clinical events that could be related to Fabry disease, and/or history of Fabry disease diagnosis) should be considered when deciding whether to initiate treatment, but is not the only factor for this decision (*statement 6K; strong consensus [1.3]*). All patients with Fabry disease (male, female, classic, late-onset) and an amenable mutation may be started on migalastat if they have evidence of Fabry-related symptoms and/or organ involvement (*statements 6A, 6B, 6D, 6E, 6F, and 6H; strong consensus [range 1.4–1.6]*). Male patients with Fabry disease and a classic, amenable mutation may be started on migalastat, even in the absence of organ involvement (*statement 6L; strong consensus [1.3]*). Female patients with Fabry disease and a classic, amenable mutation may be started on migalastat if they have ≥1 Fabry-related symptom (*statement 6I; strong consensus [1.4]*). Female patients with Fabry disease and a classic, amenable mutation should begin treatment (with either ERT or migalastat) if they have evidence of organ involvement (*statement 6C; strong consensus [1.6]*). Male and female patients with late-onset, amenable mutations should begin treatment (with either ERT or migalastat) if they have evidence of organ involvement (*statement 6G; strong consensus [1.5]*). **Treatment switch/stop** When considering whether to switch or stop treatment in patients with Fabry disease, treatment adherence, PROs, and patient choice should be considered (*statement 7A; strong consensus [1.5]*). Migalastat treatment should be continued in patients with stable or improving Fabry-related symptoms, even if they show no improvement in organ function from BL (*statement 7B; strong consensus [1.2]*). On two consecutive assessments ≥6 months apart: Relative deterioration of renal, neurological, or cardiac organ function (or architecture) compared with BL disease progression is an indication to consider switching treatment (ERT or migalastat) (*statement 7C; moderate consensus [1.0]*) Relative deterioration of symptoms alone compared with BL disease progression is not an indication to consider switching or stopping treatment (ERT or migalastat); organ involvement, PROs, and patient choice should also be considered (*statement 7D; moderate consensus [0.9]*) Patients with stabilization or improvement compared with BL disease progression in ≥1 organ should consider remaining on treatment (ERT or migalastat), even if function of another organ deteriorates (*statement 7E; moderate consensus [0.9]*).

Food and caffeine should not be consumed at least 2 h prior to and 2 h after taking migalastat to give a minimum 4 h fast (38, 39).

BL, baseline; EQ-5D, European quality of life-5 dimensions; ERT, enzyme replacement therapy; GI, gastrointestinal; HCP, healthcare professional; PRO, patient-reported outcome; and SF-36, 36-Item Short Form Health Survey.

### 4.4. Treatment decisions

Our recommendations (Table 8) provide guidance on initiating migalastat in patients with different Fabry disease phenotypes and align with previously published recommendations for the initiation of ERT (32). Regarding treatment switch or cessation, a holistic view of the patient (including clinical presentation, PROs, patient choice) should be considered on two consecutive assessments at least 6 months apart, including relative change compared with baseline disease progression. For example, if deteriorating organ function at baseline continues to deteriorate at the same rate during treatment, this may not be an indication to consider treatment switch or cessation. When making treatment decisions, healthcare practitioners should consider that organ function is unlikely to improve, as renal damage, stroke, and cardiac fibrosis are progressive and irreversible; changes from pre-treatment baseline should be considered as some patients may have significant organ damage at the start of treatment. For patients with advanced disease and irreversible damage, deterioration in one organ may not necessarily indicate lack of treatment response in other organs. Additionally, stabilization of symptoms alone does not indicate improvement in disease state, as Fabry disease is a slowly progressive disorder and may initially have only minor or no symptoms in late-onset phenotypes and females. With the implementation of individualized therapeutic goals, consideration of phenotype is therefore necessary to determine how the disease is progressing with or without treatment. More sensitive monitoring methods may be required to have confidence in disease stability.

Regarding migalastat treatment specifically, consensus was reached that decisions to continue migalastat in both male and female patients should be made regardless of the change in α-Gal A activity (see Table 6); treatment decisions should take all measured parameters into account, including all Fabry-related symptoms, signs of organ involvement, and pharmacodynamic biomarkers.

These recommendations are intended to guide the consideration of treatment switch, taking into account a holistic view of the patient, patient choice, and alternative explanations for any observed disease progression (e.g., treatment adherence). Treatment decisions should be discussed with the healthcare team and the patient themselves. Although outside the scope of this publication, a single set of guidelines would be preferable to guide treatment initiation and cessation/switch of both ERT and migalastat. Such guidelines should incorporate recommendations from this consensus survey and the guidelines of Ortiz et al. (15), and be adapted to consider the latest randomized controlled trial data, real-world evidence, and different populations, cultures, and reimbursement policies.

### 4.5. The consensus process in context

**Figure 2:**
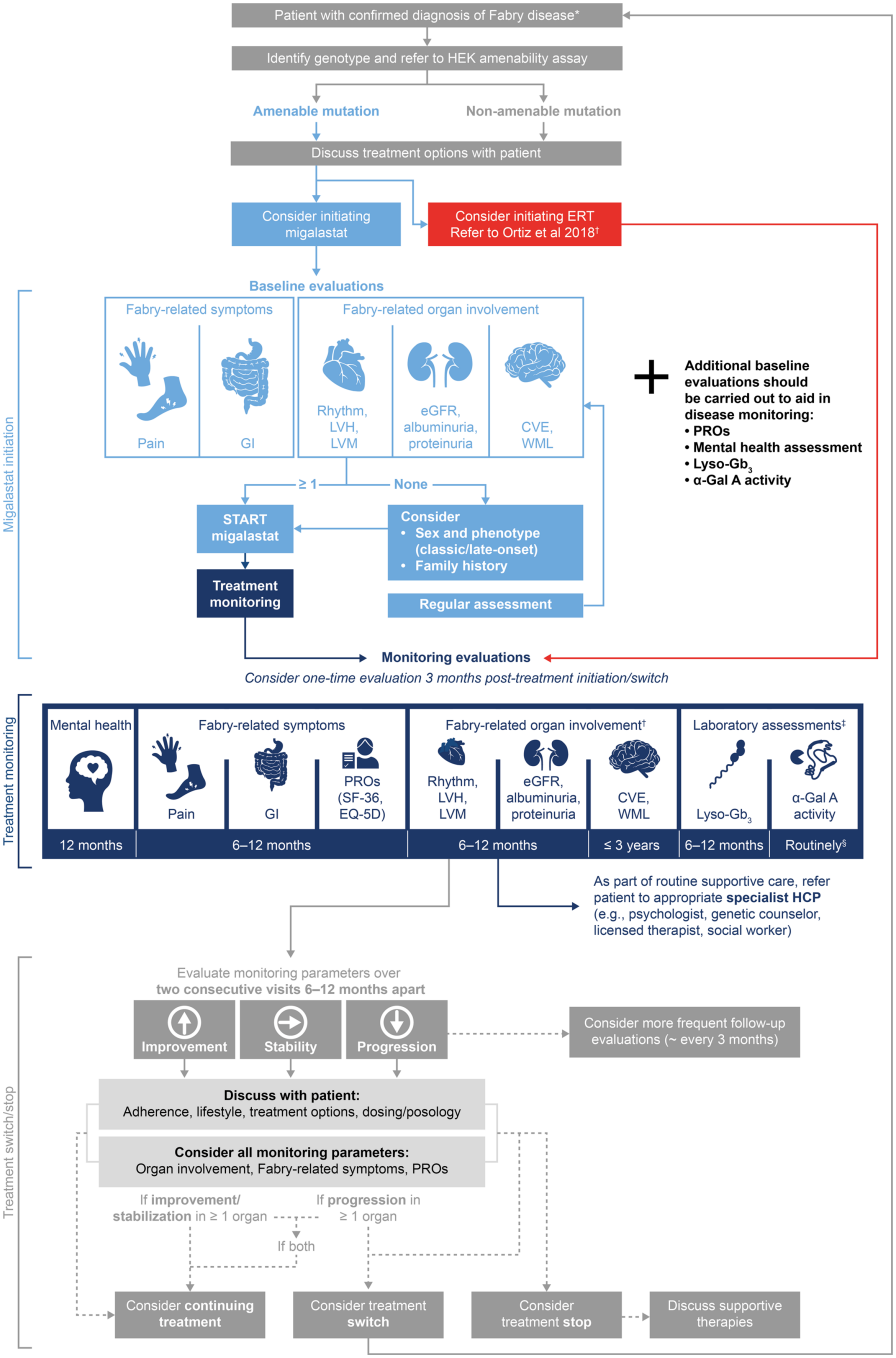
Delphi consensus recommendations for a migalastat treatment and monitoring algorithm in Fabry disease. The suggested treatment algorithm and recommendations within are not intended as a replacement for clinicians’ best judgment. ^*^Diagnosis by α-Gal A activity testing and confirmation by *GLA* genotyping. Patient eligibility for migalastat varies by country: see PI and SmPC for Galafold^®^ (38, 39); ^†^Monitoring tools for Fabry-related organ involvement are recommended in Ortiz et al. (15); ^‡^Consensus was not reached on values of lyso-Gb_3_ and α-Gal A required for treatment decisions; monitoring should be carried out for research purposes; ^§^Consensus statement recommendation 1C stated “α-Gal A activity in leukocytes should be routinely measured from migalastat initiation,” authors suggest 6–12-monthly measurements. α-Gal A, α-galactosidase A; CVE, cerebrovascular event; eGFR, estimated glomerular filtration rate; EQ-5D, European Quality of Life-5 Dimensions; ERT, enzyme replacement therapy; GI, gastrointestinal; GLA, α-galactosidase A gene; HCP, healthcare professional; HEK, human embryonic kidney; LVH, left ventricular hypertrophy; LVM, left ventricular mass; lyso-Gb_3_, globotriaosylsphingosine; PI, prescribing information; PRO, patient-reported outcome; SF-36, 36-Item Short Form Health Survey; SmPC, summary of product characteristics; and WML, white matter lesion.

This consensus process began with consideration of the patient journey in Fabry disease, focusing on decision points (including diagnosis, baseline assessments, treatment decisions, and continual monitoring) (71, 72). The panel identified how decision-making at each point in the journey impacts the patient, highlighting the importance of providing appropriate information, guidance, and support to optimize psychological as well as physical outcomes. The Delphi process led to the generation of consensus recommendations for migalastat treatment initiation, monitoring, and treatment decisions (Table 6), which are summarized in the treatment algorithm (Figure 2). These recommendations emphasize the importance of discussing treatment decisions with the patient and monitoring PROs and mental health as well as clinical symptoms. The recommendations of this publication should be considered with a focus on the patient’s psychological health during their lifelong journey with Fabry disease.

Several key gaps were identified in this publication and should be addressed in future research (Table 7); however, it also placed an emphasis on the importance of patient preference in making treatment decisions. Where research is lacking, discussion of the available evidence between healthcare professionals (HCPs) and patients can address doubts and empower the patient in the process.

### 4.6. Strengths and limitations

Strengths of this consensus process include the multidisciplinary expertise of the chairs and panel as well as the inclusion of two patients to provide a lived experience perspective. While our panel was relatively small and highly specialized, this reflects the rare and multisystemic nature of Fabry disease. Despite not representing all countries in which patients with Fabry disease are monitored or treated, our panel demonstrated long-term expertise with Fabry disease across male, female, classic and late-onset patients (Table 1).

Expert opinion (usually supplemented with a non-exhaustive literature review) is often used to develop recommendations for rare diseases such as Fabry disease, given the paucity of available data to guide a more systematic approach (15, 31, 53, 73). We used a modified Delphi method to achieve this. The Delphi method is a validated technique which has been widely used to achieve consensus among experts when limited evidence is available (52, 53), including for generation of recommendations in Fabry disease (31, 74–76). Modifications of this method are common to suit the aims of different consensus initiatives (31, 52, 73, 77, 78). We chose a previously used modification of the Delphi method in which only statements that failed to reach consensus in round 1 were reformulated and presented for panelists to rank in round 2 (52, 77, 78); the standard Delphi method presents all items to the panel for ranking in round 2, regardless of their ratings in round 1 (52). We chose this modification to streamline round 2 for focus on key areas where there was division of opinion; as our criterion for consensus was 100% agreement in round 1 (see section 2.2) and given the limited evidence base available for Fabry disease, it was deemed likely that round 1 responses reflected the true opinion of the panel.

While we based our methods on those commonly used to develop guidelines in rare diseases, they were associated with several potential limitations. Our use of a non-exhaustive rather than a systematic literature search may have meant we did not identify every relevant publication for statement development. Additionally, expert opinion has the potential for bias based on panelist experience and how representative their opinions are of wider HCPs involved in the management of patients with Fabry disease.

The majority of statements (91%) reached consensus and resulted in recommendations after two rounds of Delphi statements; however, five statements regarding α-Gal A and lyso-Gb_3_ failed to reach consensus. This highlights the difference of opinion among experts regarding the utility of these potential biomarkers for monitoring patients with Fabry disease receiving migalastat, and the recommendations herein (Tables 6, 8) should be interpreted in this context. Further research is needed regarding the utility of α-Gal A and lyso-Gb_3_ measurement in Fabry disease (see Table 7) and clinicians should consider this when utilizing these recommendations.

Additionally, this publication does not specify how organ involvement, Fabry-related symptoms, and PROs should be measured in clinical practice; we refer HCPs to the guidelines developed by Ortiz et al. (15). Lastly, the presentation of literature with round 2 Delphi statements was intended to inform the panel about their co-panelists’ rationale for round 1 responses, and therefore reformulation of statements for round 2; however, this approach could have potentially introduced bias into panelists’ round 2 responses.

### 4.7. Implications for future research

These consensus results highlight that the utility of lyso-Gb_3_ and α-Gal A as pharmacodynamic biomarkers to evaluate treatment response in patients with Fabry disease is unclear. As such, we are lacking a reliable and validated biomarker for patients with Fabry disease; this is important given the heterogenous presentation of Fabry disease, to understand and potentially predict disease progression in order to initiate timely treatment, particularly in those patients who would benefit from early treatment initiation. Further research is required to investigate current and potential biomarkers (including α-Gal A and lyso-Gb_3_ but not limited to substrate biomarkers) and determine any other potential prognostic tools. Furthermore, several therapies are in development for Fabry disease, including substrate reduction therapy, gene therapy, and combination therapies (79, 80). Future guideline updates will need to consider this developing treatment landscape while maintaining a focus on the patient journey and emotional experience.

## 5. Conclusion

The heterogenous clinical presentation of patients with Fabry disease necessitates detailed guidelines that consider phenotype and multidisciplinary monitoring assessments, to recommend appropriate treatment and monitoring decisions (15, 80). Migalastat is an addition to the treatment armamentarium that has not yet been reflected in all treatment guidelines (15, 81). This consensus process aimed to complement and build on previously published guidelines (15) by addressing treatment initiation and management in patients receiving migalastat, as well as highlighting the importance of the patient journey (Figure 2). These recommendations comprise up-to-date guidance collected during a modified Delphi process involving 14 leading experts and two patient advocates. We hope that this publication will lead to the provision of consistent high-quality care with a shared decision-making process considering a holistic view of the patient’s experience, including clinical presentation, PROs, and patient preference.

## Data availability statement

The original contributions presented in the study are included in the article/Supplementary material, further inquiries can be directed to the corresponding author.

## Ethics statement

Ethical review and approval was not required for the study on human participants in accordance with the local legislation and institutional requirements. The patients/participants provided their written informed consent to participate in this study.

## Author contributions

The co-chairs, DB, RH, and DH, provided expert clinical insight throughout this consensus study and manuscript development. PA, SA, YHC, RG, SaK, StK, OL, DMN, IO, JP, PR, RT, and CT were voting members of the panel and provided expert input in two Delphi surveys and on the interpretation of the findings. All authors contributed to the article and approved the submitted version.

## Funding

This study received funding from Amicus Therapeutics. The funder had the following involvement with the study: financial support for third-party administrative, project management, medical writing, and editorial support. The funder was not involved in the study design, collection, analysis, or interpretation of data.

## Conflict of interest

DB is a member of the Fabry Registry Advisory Board (sponsored by Sanofi) and the follow ME Fabry Pathfinders registry (sponsored by Amicus Therapeutics), is a consultant and has acted as a speaker for Amicus Therapeutics, Sanofi, and Takeda, and has been an investigator in clinical trials sponsored by Amicus Therapeutics, Idorsia, Sanofi, and Takeda. These activities have been monitored and found to be in compliance with the conflict of interest policies at Sacré-Coeur Hospital in Montreal and the University of Montreal. RH is a member of the Fabry Registry Advisory Board (sponsored by Sanofi) and the follow ME Fabry Pathfinders registry (sponsored by Amicus Therapeutics), is a consultant for Amicus Therapeutics, Chiesi, Sanofi, and has been an investigator in clinical trials sponsored by Amicus Therapeutics, Chiesi, Idorsia, Protalix Biotherapeutics, Sangamo Therapeutics, Sanofi, and Takeda. These activities have been monitored and found to be in compliance with the conflict of interest policies at Cincinnati Children’s Hospital Medical Center. RH is also chair of the Medical Advisory Committee and a speaker for the National Fabry Disease Foundation and is an advisor and a speaker for the Fabry Support and Information Grozup. DH is a consultant and has acted as a speaker for Takeda, Sanofi, Amicus Therapeutics, Inc., Idorsia, Freeline, and Protalix. Consultancy fees and speaker honoraria are administered via University College London Consultants and used in part to support research in lysosomal storage disorders. PA receives grant/research support from Takeda, and speaker/travel honoraria from Takeda, Sanofi-Genzyme, BioMarin, Ultragenyx, Alexion, Amicus Therapeutics, and Chiesi. SA is employed by Premier Physicians Group Health (Fort Worth, Texas, United States), and serves as a speaker and/or consultant for Veloxis, Sanofi, Alexion, CareDx, Natera, and Amicus Therapeutics. YHC has received research support from Sanofi and Biogen, and has received consulting fees/honoraria/travel reimbursement from Sanofi, Amicus, Audentes, Biogen, Novartis, Roche, PTC therapeutics, AskBio, BioMarin, and Takeda. RG is a member of the Fabry Outcome Survey (sponsored by Takeda) and followME (sponsored by Amicus) steering committees, and has received research support from Allievex, Amicus, Avrobio, Azafaros, Chiesi, Cyclo, Idorsia, Janssen, JCR, Novartis, Paradigm, PassageBio, PTC, RegenxBio, Sanofi, Takeda, and Ultragenyx, consulting fees from Abeona, Amicus, Avrobio, Azafaros, BioMarin, Chiesi, Cyclo, DASA, Denali, Inventiva, Janssen, JCR, Novartis, Pfizer, PTC, RegenxBio, Sanofi, Sigilon, Sobi, Takeda, and Ultragenyx, and speaker honoraria from Amicus, Azafaros, Chiesi, Janssen, JCR, Novartis, Pfizer, PTC, RegenxBio, Sanofi, Takeda, and Ultragenyx. SaK is a patient advocate with ongoing responsibilities as a Fabry Champion (Amicus Therapeutics), Patient Advisory Council Member (Lightship) and Community Ambassador (TRENDCommunity), and has served on a patient advisory board and received honoraria from Chiesi, and received speaker honoraria from Sangamo Therapeutics. StK has served as a consultant for Amicus Therapeutics, has received speaker honoraria and travel fees from Amicus Therapeutics and Sanofi, and has been an investigator in clinical trials sponsored by Aytu BioPharma, Idorsia, Incyte, and Ipsen. These activities have been monitored and found to be in compliance with the conflict of interest policies of the University of Pennsylvania. OL has received travel grants and speaker honoraria from Amicus, Chiesi, Sanofi Genzyme, and Takeda. DMN is a member of Fabry Registry Advisory Board (sponsored by Takeda and Sanofi) and a consultant for Amicus Therapeutics, Sanofi, and Takeda. He has also been an investigator in clinical trials sponsored by Protalix Biotherapeutics, Sanofi, Takeda, and 4DMT. IO has received research grants from BMS-Myokardia, Cytokinetics, Boston Scientific, Amicus, Sanofi Genzyme, Shire Takeda, Menarini International, Bayer, Chiesi, and Tenaya and has participated in advisory boards with BMS-Myokardia, Cytokinetics, Amicus, Sanofi Genzyme, Chiesi, Tenaya, and Rocket Pharma. JP has received honoraria from Amicus Therapeutics, Sanofi Genzyme, Idorsia, Freeline tx, and Biosidus, and consulting fees from Sanofi Genzyme, Biosidus, and Amicus Therapeutics. PR serves as a Patient Care Advocate for Fabry disease on the Patient Advisory Board for Amicus Therapeutics. RT has received consulting fees from Amicus Therapeutics, Takeda, Chiesi, and Sanofi Genzyme, and honoraria for lectures, presentations, speaker bureaus, manuscript writing, or educational events from Amicus Therapeutics, Takeda, Chiesi, and Sanofi Genzyme, and is a council member of the European Renal Association (ERA). CT has, on behalf of Haukeland University Hospital, served as consultant for Sanofi, Amicus, Chiesi, Freeline, and Acelink, participates as investigator in clinical studies initiated by Sanofi, Protalix, Idorsia, and Freeline, and has received speaker honoraria from Sanofi, Amicus, Takeda, and Chiesi. All honoraria received go to Haukeland University Hospital.

## Publisher’s note

All claims expressed in this article are solely those of the authors and do not necessarily represent those of their affiliated organizations, or those of the publisher, the editors and the reviewers. Any product that may be evaluated in this article, or claim that may be made by its manufacturer, is not guaranteed or endorsed by the publisher.
