# Supplementary material for: Consensus recommendations for the treatment and management of patients with Fabry disease on migalastat: a modified Delphi study
Source: Front Med (Lausanne). 2023 Sep 1;10:1220637. doi: 10.3389/fmed.2023.1220637 (PMC10505750; doi:10.3389/fmed.2023.1220637)
Supplement: Supplementary file 1 [file Data_Sheet_1.docx]

Supplementary Material

Consensus recommendations for the treatment and management of patients with Fabry disease on migalastat: a modified Delphi study

Daniel G. Bichet, Robert J. Hopkin, Patrício Aguiar, Sridhar R. Allam, Yin-Hsiu Chien, Roberto Giugliani, Staci Kallish, Sabina Kineen, Olivier Lidove, Dau-Ming Niu, Iacopo Olivotto, Juan Politei, Paul Rakoski, Roser Torra, Camilla Tøndel, Derralynn A. Hughes*

*** Correspondence:** Professor Derralynn A. Hughes: rmgvdah@ ucl.ac.uk

# Appendix A. Literature review

**A.1 Non-exhaustive literature search to identify guidelines or management recommendations for patients with Fabry disease receiving migalastat**

Before the initial meeting between the consensus committee in 2021, a non-exhaustive PubMed literature search was performed by the third-party administrator to identify guidelines or management recommendations for patients with Fabry disease receiving migalastat. The findings of the literature search were shared with the consensus committee during the meeting.

The following search string was used and limited to publications since 2018, when migalastat was approved: ((Fabry disease OR alpha-galactosidase A deficiency OR α-galactosidase A deficiency OR Anderson-Fabry disease OR angiokeratoma corporis diffusum OR diffuse angiokeratoma OR GLA deficiency)[Title/Abstract] OR Fabry disease [MeSH Major Topic]) AND (guideline OR guidelines OR consensus OR statement OR statements OR recommendation OR recommendations OR algorithm OR algorithms OR management)[Title/Abstract]))

Due to the nature of local guidelines for rare diseases, which are often in local languages and may not be available via PubMed searches, an additional grey literature search was conducted using the Google search string: ((Fabry disease OR alpha-galactosidase A deficiency OR α-galactosidase A deficiency OR Anderson-Fabry disease OR angiokeratoma corporis diffusum OR diffuse angiokeratoma OR GLA deficiency) AND (guideline OR guidelines OR consensus OR statement OR statements OR recommendation OR recommendations OR algorithm OR algorithms OR management)). Additional webpages relevant to rare diseases were searched: [www.orpha.net](http://www.orpha.net/); [www.eurordis.org](http://www.eurordis.org/); [www.rarediseases.org](http://www.rarediseases.org/); <https://www.rarediseasesnetwork.org/>; [www.nice.org.uk](http://www.nice.org.uk/); [www.rarediseases.info.nih.gov](http://www.rarediseases.info.nih.gov/). The full text of all results was screened for specific guidelines/recommendations concerning migalastat.

**A.2 Non-exhaustive literature search to inform reformulation of statements at round 2:**

Titles and abstracts of English language articles published over a period of 15 years (2007–2022) were searched and screened for relevance to the key areas covered by the round 1 statements. Case reports and systematic reviews/meta-analyses were included, and opinion-based reviews and animal model studies were excluded. Additional publications were identified in free-text responses by the panelists during round 1, and results from the initial literature search for guidelines (see section A.1) were also considered. Abstracts and full text (where available) of relevant studies were reviewed and used to guide reformulation of the statements for round 2. A summary of relevant studies was presented to the panel along with round 2 Delphi statements to provide context for reformulation of each statement.

The following search strings were conducted: (Fabry OR Anderson-Fabry[Title]) AND ((a-Gal A activity[Title/Abstract]) OR (alpha-galactosidase A activity[Title/Abstract]) OR (alpha-Gal A activity[Title/Abstract]) OR (enzyme activity[Title/Abstract])) AND ((migalastat) OR (dgj) OR (1-deoxygalactonijirimycin) OR (galafold)); (Fabry OR Anderson-Fabry[Title]) AND (lyso-Gb3 OR globotriaosylsphingosine OR GL-3 OR GL3 OR lysoGb3[Title/Abstract]); (Fabry OR Anderson-Fabry[Title]) AND (mental health OR psychiatric OR psychological OR quality of life[Title/Abstract]).

# Appendix B. Round 1 virtual Delphi survey

The virtual survey was split into seven sections covering the following topics:

- α-Galactosidase A enzyme (α-Gal A) testing
- Globotriaosylsphingosine (lyso-Gb_3_) testing
- Patient perspective
- Treatment monitoring for male and female patients and different phenotypes
- Mental health
- Patient-reported outcomes (including pain and gastrointestinal [GI] symptoms)
- Switch/stop treatment

The structure of the round 1 Delphi survey for each statement was as follows:
**Indicates required field*

**Statement***

**Please rate the above statement***

( ) Strongly agree ( ) Agree ( ) Disagree ( ) Strongly disagree

**Rationale/supporting evidence**

____________________________________________

____________________________________________

____________________________________________

____________________________________________

**If you DISAGREE or STRONGLY DISAGREE, please provide the reasoning why**

____________________________________________

____________________________________________

____________________________________________

____________________________________________

Round 1 statements were as follows:

**Section 1: α-Gal A testing**

1. α-Gal A enzyme activity testing in leukocytes should be undertaken at diagnosis and at first follow-up
2. In patients receiving migalastat, subsequent α-Gal A enzyme testing in leukocytes should be performed if there is a change in clinical status
3. In patients receiving migalastat, subsequent α-Gal A enzyme testing in leukocytes should be performed if there is a change in signs of decline
4. Measurement of α-gal A activity in leukocytes is an important way of confirming the biological activity of migalastat
5. To continue migalastat, it is reasonable to expect α-gal A activity to increase to at least 10% of normal levels
6. To continue migalastat, it is reasonable to expect α-gal A activity to increase by at least 10% from baseline
7. To continue migalastat, it is reasonable to expect α-gal A activity to increase to at least 20% of normal levels
8. To continue migalastat, it is reasonable to expect α-gal A activity to increase by at least 20% from baseline
9. To continue migalastat, it is reasonable to expect α-gal A activity to increase to at least 30% of normal levels
10. To continue migalastat, it is reasonable to expect α-gal A activity to increase by at least 30% from baseline
11. Measuring α-gal A activity in female patients is helpful in the decision-making process on whether to continue migalastat
12. Migalastat should be continued in both male and female patients who are showing an improvement in disease-related symptoms, regardless of the change in α-gal A activity
13. Migalastat should be continued in both male and female patients with stable or improved organ function, regardless of any change in the α-gal A activity

**Section 2: Lyso-Gb_3_ testing**

1. Lyso-Gb_3_ analysis should be performed in all patients at diagnosis
2. Lyso-Gb_3_ analysis should be performed every 6 months in patients receiving treatment with enzyme replacement
3. Lyso-Gb_3_ analysis should be performed every 6 months in patients receiving treatment with migalastat
4. Lyso-Gb_3_ is a reliable pharmacodynamic biomarker that can be used to confirm the biological activity of migalastat
5. In treatment-naïve male patients, any decrease in lyso-Gb_3_ from baseline is sufficient to confirm the biological activity of migalastat
6. In treatment-naïve female patients, any decrease in lyso-Gb_3_ from baseline is sufficient to confirm the biological activity of migalastat
7. In treatment-naïve male patients, failure to reduce lyso-Gb_3_ by ≥ 30% from pre-treatment baseline is an indication to change therapy after 12 months of migalastat treatment
8. In treatment-naïve female patients, failure to reduce lyso-Gb_3_ by ≥ 30% from pre-treatment baseline is an indication to change therapy after 12 months of migalastat treatment
9. In treatment-naïve male patients, failure to reduce lyso-Gb_3_ by ≥ 50% from pre-treatment baseline is an indication to change therapy after 12 months of migalastat treatment
10. In treatment-naïve female patients, failure to reduce lyso-Gb_3_ by ≥ 50% from pre-treatment baseline is an indication to change therapy after 12 months of migalastat treatment
11. In treatment-naïve male patients, failure to reduce lyso-Gb_3_ by ≥ 70% from pre-treatment baseline is an indication to change therapy after 12 months of migalastat treatment
12. In treatment-naïve female patients, failure to reduce lyso-Gb_3_ by ≥ 70% from pre-treatment baseline is an indication to change therapy after 12 months of migalastat treatment
13. In treatment-experienced male patients (switched from enzyme replacement therapy [ERT]), stable lyso-Gb_3_ levels, relative to lyso-Gb_3_ levels while receiving ERT, are sufficient to confirm the biological activity of migalastat
14. In treatment-experienced female patients (switched from ERT), stable lyso-Gb_3_ levels, relative to lyso-Gb_3_ levels while receiving ERT, are sufficient to confirm the biological activity of migalastat
15. In treatment-experienced male patients (switched from ERT), failure to reduce lyso-Gb_3_ by ≥ 30% relative to lyso-Gb_3_ levels while receiving ERT, is an indication to change therapy after 12 months of migalastat treatment
16. In treatment-experienced female patients (switched from ERT), failure to reduce lyso-Gb_3_ by ≥ 30% relative to lyso-Gb_3_ levels while receiving ERT, is an indication to change therapy after 12 months of migalastat treatment
17. In treatment-experienced male patients (switched from ERT), failure to reduce lyso-Gb_3_ by ≥ 50% relative to lyso-Gb_3_ levels while receiving ERT, is an indication to change therapy after 12 months of migalastat treatment
18. In treatment-experienced female patients (switched from ERT), failure to reduce lyso-Gb_3_ by ≥ 50% relative to lyso-Gb_3_ levels while receiving ERT, is an indication to change therapy after 12 months of migalastat treatment
19. In treatment-experienced male patients (switched from ERT), failure to reduce lyso-Gb_3_ by ≥ 70% relative to lyso-Gb_3_ levels while receiving ERT, is an indication to change therapy after 12 months of migalastat treatment
20. In treatment-experienced female patients (switched from ERT), failure to reduce lyso-Gb_3_ by ≥ 70% relative to lyso-Gb_3_ levels while receiving ERT, is an indication to change therapy after 12 months of migalastat treatment
21. Current migalastat treatment should be continued in any patient showing a symptomatic response (response in symptoms), regardless of the change in lyso-Gb_3_
22. Current migalastat treatment should be continued in any patient showing stable or improved organ function regardless of any change in lyso-Gb_3_
23. Following any increase in lyso-Gb_3_ from baseline, where there has been no change in treatment, patients should be asked about their adherence to migalastat
24. Adherence and compliance with migalastat should be discussed with the patient in a systematic way, reviewing the patient’s current dosing and posology

**Section 3: Patient perspective**

1. Patients should be given an overview of all suitable available therapies
2. Patients should be encouraged to be involved in shared decision-making when starting, changing, or stopping therapy
3. Patient choice should be taken into consideration in regard to any decision to start therapy, change therapy, or stop therapy

**Section 4: Treatment monitoring**

1. Migalastat and enzyme replacement therapy should follow the same guidelines and recommendations when it comes treatment initiation
2. Migalastat and enzyme replacement therapy should follow the same guidelines and recommendations when it comes treatment cessation
3. Family history is an important factor when deciding whether to initiate therapy
4. Male patients may be started on migalastat if they have evidence of Fabry-related symptoms and an amenable mutation
5. Female patients may be started on migalastat if they have evidence of Fabry-related symptoms and an amenable mutation
6. Male patients may be started on migalastat if they have evidence of organ involvement and an amenable mutation
7. Female patients may be started on migalastat if they have evidence of organ involvement and an amenable mutation
8. Patients with classic or late-onset Fabry disease may both be started on migalastat if they have evidence of Fabry-related symptoms and an amenable mutation
9. Patients with classic or late-onset disease may both be started on migalastat if they have evidence of organ involvement and an amenable mutation
10. Male patients may be started on migalastat in the presence of a classic Fabry variant, amenable to migalastat, even in the absence of organ involvement
11. Female patients may be started on migalastat in the presence of a classic Fabry variant, amenable to migalastat, if they have at least one Fabry-related symptom
12. Female patients with classic variants that are amenable to migalastat should commence therapy if they have evidence of organ involvement
13. Male and female patients with late-onset mutations that are amenable to migalastat should commence therapy if they have evidence of organ involvement

**Section 5: Mental health**

1. All patients with Fabry disease should be evaluated for mental health by a healthcare professional, using a validated screening tool
2. All patients with Fabry disease should be assessed for mental health at baseline and at 6- to 12-month intervals by a healthcare professional, using a validated screening tool
3. All patients should be referred to a counselor or psychologist as appropriate, based on the results of the mental health assessment

**Section 6: Patient-reported outcomes (PROs; including pain and GI symptoms)**

1. All patients with Fabry disease should be evaluated for pain and GI symptoms at baseline (i.e., prior to treatment initiation)
2. All patients with Fabry disease should have interval evaluations with PROs every 3 months
3. All patients with Fabry disease should have interval evaluations with PROs every 3–6 months
4. All patients with Fabry disease should have interval evaluations with PROs every 6 months
5. All patients with Fabry disease should have interval evaluations with PROs every 6–12 months
6. All patients with Fabry disease should have interval evaluations with PROs annually
7. All patients with Fabry disease should have interval evaluations with PROs every 2 years

**Section 6: Switch/stop treatment**

1. All patients should have an evaluation of renal, cardiac, and neurological function at baseline and every 6 months thereafter
2. All patients should undergo an evaluation of Fabry-related symptoms at baseline and on an annual basis thereafter
3. Deterioration of renal, neurological, or cardiac organ function (or architecture) on two consecutive readings at least 6 months apart is an indication to consider switching ERT or migalastat therapy due to lack of efficacy
4. Deterioration of symptoms on two consecutive readings at least 6 months apart is an indication to consider switching or stopping ERT or migalastat therapy
5. ERT therapy should not be stopped in an individual where one or more organ functions are stable or improving, even if another organ function has demonstrated deterioration
6. Therapy with migalastat should not be stopped in an individual where symptoms are stable or improving even if organ function has not demonstrated improvement relative to the patient’s history

**Appendix C. Round 2 virtual Delphi survey: structure**

All round 2 Delphi statements are presented in Tables 2–5 of the article. The structure of the round 2 Delphi survey for each statement was as follows:

**Round 1 statement (and % of agree/strongly agree/disagree/strongly disagree)**

| Summarized free-text “rationale/supporting evidence” for “agree” or “strongly agree” answers from round 1 |
| --- |

| Summarized free-text “reasoning” for “disagree” or “strongly disagree” answers from round 1 |
| --- |

**Reformulated statement for round 2:** __________________________________________

Rationale for reformulation: ___________________________________________________

( ) Strongly agree ( ) Agree ( ) Neither agree nor disagree ( ) Disagree ( ) Strongly disagree

| Optional free-text field for general comments: |
| --- |
| *Type comments here* |

A selection of literature, based on Delphi round 1 survey “rationale/supporting evidence” free-text panelist responses, relevant to round 2 statement was presented with the statement.
